# Risk of developing a second primary cancer following a renal cell carcinoma: a systematic review and meta-analysis

**DOI:** 10.1007/s11255-025-04794-7

**Published:** 2025-09-18

**Authors:** Samuel Tundealao, Praise Okunlola, Tolulope Titiloye, Bolatito Mayungbo, Abiodun Adegbesan, Anusha Sajja, Olajumoke Olarewaju

**Affiliations:** 1 Department of Biostatistics and Epidemiology, Rutgers School of Public Health, 683 Hoes Lane, Piscataway, NJ 08854 USA; 2 Rutgers Cancer Institute, New Brunswick, NJ USA; 3 Faculty of Dentistry, College of Medicine, University of Ibadan, Ibadan, Oyo Nigeria; 4 Mental Health Association of Morris and Sussex, Parsippany, NJ USA; 5 Menzies Institute for Medical Research, University of Tasmania, Hobart, Australia; 6 School of Public Health, University of Texas Health Science Center at Houston, Houston, TX USA; 7 Department of Health, Behavior, and Society, Johns Hopkins Bloomberg School of Public Health, Baltimore, MD USA

**Keywords:** Renal cell carcinoma, Kidney cancer, Second primary cancer, Multiple malignancies

## Abstract

**Purpose:**

This study synthesizes previous studies to investigate the risk of second primary cancer (SPC) in patients with renal cell carcinoma (RCC) compared with the general population.

**Methods:**

Following the PRISMA guidelines, four databases were searched for relevant articles without date limits. Studies were included if they reported the standardized incidence ratio (SIR) for SPCs in patients with RCC compared with the general population. Random-effects model employing the Der Simonian and Laird method was used to pool the SIR by SPC sites. Effect heterogeneity was assessed using *I*^2^.

**Results:**

Twenty-seven retrospective cohort studies published between 1993 and 2024 were included in the study. The overall risk of developing any SPCs was higher among RCC patients (pooled SIR = 1.32, CI 1.19–1.48). The risk of several SPCs was significantly higher in patients with RCC compared with the general population, including cancers of the contralateral kidney (3.68, CI 1.85–7.24), thyroid (3.05, CI 2.57–3.62), urinary bladder (2.15, CI 1.40–3.30), small intestine (1.98, CI 1.01–3.91), leukemia (1.86, CI 1.28–2.70), pancreas (1.64, CI 1.22–2.19), NH lymphoma (1.55, CI 1.21–1.98), melanoma (1.47, CI 1.15–1.88), prostate (1.44, CI 1.21–1.71), and colorectal (1.25, CI 1.10–1.43), bone (2.31, CI 1.25–4.27), endocrine system (4.33, CI 3.28–5.74) and brain/nervous system (2.20, CI 1.25–3.88).

**Conclusion:**

Patients with RCC have an increased risk of SPCs compared with the general population. These findings highlight the need to develop strategies for the management of SPCs in these patients.

**Supplementary Information:**

The online version contains supplementary material available at 10.1007/s11255-025-04794-7.

## Introduction

The World Health Organization’s International Agency for Research on Cancer (IARC) reported a global incidence of 434,840 cases of kidney cancer in 2022, with an estimated mortality rate of 36% [1]. Developing from the renal tubular epithelial cells, renal cell carcinoma (RCC) is the most predominant kidney cancer, accounting for over 90% of cases [2]. Among the histological subtypes of RCC, clear cell carcinoma is the most common, constituting about 75% of its presentations [3]. In 2023, the incidence of RCC in the United States (US) was reported to be over 80,000 cases, ranking it as the sixth most common malignancy in males, and the ninth in females, with a mortality rate of about 17% [4]. In developed countries all over the world, the mortality rate of RCC is gradually declining owing to early diagnosis and implementation of prompt therapy [2]. As survival rates improve, complications such as second primary cancers (SPCs) have become an increasing concern [2].

The International Association of Cancer Registries (IACR) and the IARC define SPCs as a de novo and separate cancer developing in an individual with a previous primary cancer [5]. These second primary cancers are histologically different from the primary cancers and develop by means other than the metastasis, recurrence, or extension of the primary cancer [5]. A 2013 study on the incidence of second primary cancers in RCC patients found a notable standardized incidence ratio (SIR) of 1.18 [6]. A 2024 Danish study by Bengsten et al. showed an even higher SIR of 2.3 within a 1-year follow-up period [7]. The literature has linked the development of SPC to multiple factors such as cancer treatment, e.g., radiotherapy, genetic factors, viral infections, and risk factors such as alcohol and tobacco use [8].

Some studies have highlighted the risk of development of SPC following RCC, but most of these were carried out among the US, Swedish, and Danish populations, thus not providing a complete estimate of its risk among diverse populations [6, 9–11]. Furthermore, certain studies evaluated the risk of SPCs in individuals diagnosed over 20 years ago and do not consider the thousands of people diagnosed more recently. Despite the increasing incidence of SPCs in cancer survivors, pooled data on their occurrence following RCC remains limited. To date, no systematic review and meta-analysis exists that comprehensively examines and pools the risk of SPCs among patients diagnosed with RCC. Therefore, this systematic review and meta-analysis aims to evaluate the risk of SPCs in RCC patients compared with the general population.

## Methods

This study was conducted following the Preferred Reporting Items for Systematic Reviews and Meta-Analyses (PRISMA) reporting guideline. The study’s protocol was also preregistered in PROSPERO (CRD420251072650).

### Search strategy and sources

In consultation with a health sciences librarian, we designed and conducted the database search. PubMed/MEDLINE, Scopus, Web of Science, and Embase databases were searched. The search strategy was designed to identify studies that report the risk or standardized incidence of SPCs among RCC patients. The searches included terms for risk/incidence, second primary cancer/malignancy, and RCC (eTable 1, Supplement). Searches were not restricted by publication year. The initial search was conducted on May 1st, 2025, and updated on June 26, 2025.

### Eligibility and study selection

Articles were eligible for inclusion in this review if (1) they reported the risk of SPCs in patients with RCC using the standardized incidence ratio (SIR). We restricted to studies that reported SIR estimates because SIR is an indirect standardization method that adjusts for age, sex, and other characteristics. No restrictions were applied to age, gender, comorbidities, duration, or location of the study, nor method of reporting cancer diagnoses, (2) were published in peer-reviewed journals; and (3) were published in the English language.

Articles were excluded if (1) they did not report overall or cancer site-specific risk of SPCs in RCC patients; (2) they reported synchronous second or multiple cancer; (3) they reported the risk of SPC in patients with nephroblastoma (Wilms’ tumor) and renal pelvis; (4) they were meta-analysis/systematic review, book chapters, Master’s, doctorates, editorials, reviews; (5) were published in languages other than English; and (6) they had overlapping populations, data source/registry and time periods. We excluded the older studies when follow-up periods fell entirely within the timeframe of a newer study using the same database/registry, and the recent study covered a broader or updated timeframe and reported on similar SPC risks.

Two investigators (S.T. and P.O.) independently evaluated the titles, abstracts, and full texts to identify papers that satisfied the inclusion criteria. Discrepancies were reconciled through consensus between the pair or a third reviewer. The screening and research selection process utilized Covidence software.

### Data extraction

Two investigators (S.T. and P.O.) independently extracted data using the predefined extraction form created on the Covidence software. The data that were extracted included the title of the study, the first author’s name, the country where the study was conducted, the study design, the study setting, the study population characteristics (total sample size, mean age, and patients’ sex), follow-up duration, and outcomes (SIR per cancer type or SIR for any SPC). Diagnosis and confirmation of RCC and SPCs were done according to the criteria of each study. Data extraction forms were sent to the corresponding authors of the included studies, when necessary, with a request to provide missing data, if applicable. Disagreements were resolved through consensus between the pair of raters or by a third rater.

### Outcome

The main outcome assessed in this meta-analysis was the risk or incidence of SPCs after RCC, measured using the SIR. In each study, the SIR was calculated as the ratio of observed cases of SPCs to the expected cases of the cancers in the general population. If the study reported only the observed and expected cases, we calculated the SIR and corresponding 95% CI using the Mid-P exact test on the OpenEpi software [12]. Table 1 provides a comprehensive explanation of how each study calculated the number of expected cases.

**Table 1:** Characteristics and quality of included studies

Study ID	Study type	Country	Data source	Time frame	Sample size (RCC)	SPC reported	Calculation of expected cases	Newcastle–Ottawa scale rating
Anderson [16]	Retrospective cohort study	USA	Institutional Tumor Registry Database at MD Anderson Cancer Center	1988–1995	2099	NH lymphoma	United States population-based cancer statistics (Surveillance, Epidemiology and End Results Program data) were used to arrive at the expected age adjusted incidence of co-occurrence of these diagnose	4
Barocas [17]	Retrospective cohort study	USA	Surveillance Epidemiology and End Results (SEER)	1973–1996	Not reported for RCC	Prostate	The expected number of second cancers was obtained by multiplying the incidence rates by the number of person-years at risk in each category, with these products summed over the different age groups, genders, and calendar years	8
Beisland [18]	Retrospective cohort study	Norway	Cancer Registry of Norway	1987–1993	1425	Any cancer, urinary bladder, brain, nervous system, breast, lungs, melanoma, NH lymphoma, prostate, stomach, and colorectal	The expected number of cases was estimated by assuming that the patients in the cohort had the same cancer incidence as prevailed in the general population of Norway. Using the Main Database of the Cancer Registry of Norway, tumor site-, gender-, period- and age-specific rates were combined with the person-years at risk, the last being accumulated for each person starting with date of diagnosis of RCC and ending with date of death, emigration or 31 December 2002, whichever was soonest	8
Bengtsen [7]	Retrospective cohort study	Denmark	Danish Cancer Registry	1995–2019	14,549	Any cancer, renal pelvis, urinary bladder, brain, nervous system, breast, female genitalia, lungs, melanoma, multiple myeloma, NH lymphoma, leukemia, liver, pancreas, male genitalia, stomach, small intestine, colorectal, oropharynx, endocrine and mesothelioma	Expected number of cancers was calculated based on national cancer incidence rates, according to sex, age (5-year groups), and calendar year (5-year groups)	8
Boakye [19]	Retrospective cohort study	USA	Surveillance Epidemiology and End Results (SEER)	2000–2014	117,278	Any cancer	The number of expected SPMs was calculated for a non-cancer patient cohort of identical age, sex, ethnic group, and time period. The expected numbers of second cancers was estimated by multiplying sex-, age-, ethnic group-, and calendar year-specific SEER cancer incidence rates with the accumulated person-years at risk	8
Chakraborty [6]	Retrospective cohort study	USA	Surveillance Epidemiology and End Results (SEER)	1973–2006	Not reported for RCC	Any cancer, kidney, renal pelvis, urinary bladder, brain, nervous system, breast, female genitalia, lungs, melanoma, multiple myeloma, NH lymphoma. Leukemia, liver, male genitalia, oropharynx, endocrine, bone, soft tissue and mesothelioma	Expected were the number subsequent cancers at the same site in the general population	8
Chen [9]	Retrospective cohort study	Sweden	Swedish Family Cancer Database	1990–2010	14,267	Kidney, urinary bladder, brain, nervous system, breast, uterine, ovarian, lungs, melanoma, multiple myeloma, NH lymphoma, leukemia, esophagus, liver, pancreas, prostate, stomach, small intestine, colorectal, thyroid, oropharynx, endocrine, bone and soft tissue	The expected numbers of second cancer in RCC survivors were calculated from the strata-specific first cancer incidence rate in the Swedish general population, multiplied by the corresponding person-years for second cancer in RCC survivors	8
Chen [20]	Retrospective cohort study	Sweden	Swedish Cancer Registry	1958–2012		Mesothelioma	The expected numbers of SPC were calculated from the strata-specific first same cancer incidence rates in the Swedish general population, multiplied by the corresponding person-years for second cancer	7
Choi [21]	Retrospective cohort study	Korea	Health Insurance Review and Assessment database of South Korea	2009–2019	5221	Any cancer	The Korean National Cancer Statistics 2015, the middle year of the cohort were used as the expected cancer rates	6
Crocetti [22]	Retrospective cohort study	Italy	13 population-based cancer registries	1989–1995	Not reported for RCC	Urinary bladder and colorectal	The expected number was calculated by multiplying the age-, gender-, 5-year period-, site- and registry-specific incidence rates by the same categories of person-year	6
Crocetti [23]	Retrospective cohort study	Italy	28 population-based Italian cancer registries	1998–2012		Thyroid	The expected number of cancer cases was computed by multiplying the cumulative person-years of observation by the specific incidence rates for the strata in which person-years were distributed	6
Czene [24]	Retrospective cohort study	Sweden	Swedish Family Cancer Database	1958–1998	23,137	Any cancer, urinary bladder, brain, nervous system, breast, cervix, uterine, ovarian, lungs, melanoma, NH lymphoma, leukemia, liver, pancreas, prostate, stomach, colorectal, oropharynx, and endocrine	The expected numbers of cancers were obtained by assuming that these persons experienced the same cancer incidence as prevailed in the corresponding general population in the Database	8
Jegu [25]	Retrospective cohort study	France	Ten French cancer registries	1989–2004	Not reported for RCC	Any cancer and kidney	The number of expected cancers was computed by multiplying person-years at risk (allocated by gender, attained age, year of follow-up and first cancer region of diagnosis) with corresponding first cancer incidence rates from the general population	6
Joung [26]	Retrospective cohort study	Korea	Korean Central Cancer Registry	1993–2013	40,347	Any cancer, kidney, renal pelvis, urinary bladder, breast, cervix, female genitalia, gallbladder, lungs, melanoma, leukemia, esophagus, prostate, male genitalia, stomach, thyroid, oropharynx, bone and soft tissue	Cancer incidence rates were calculated for each type of cancer, by sex, age and calendar year, and were multiplied by the accumulated person-years at risk to estimate the expected number of subsequent cancers for each stratum	8
Kamath [27]	Retrospective cohort study	USA	Surveillance Epidemiology and End Results (SEER)	2000–2015	Not reported for RCC	Pancreas	The expected number of cases was calculated by multiplying age- and sex-specific population cancer incidence rates with the age- and sex-specific person-years at risk among the cohort	8
Kang [28]	Retrospective cohort study	Korea	Korea Central Cancer Registry	1993–2017		Pancreas	The mid-year population reported by Statistics Korea was used as a general reference population in this study	6
Kim [29]	Retrospective cohort study	USA	Institutional Tumor Registry Database at The University of Texas MD Anderson Cancer Center (MDACC)	1980–2005	7597	Melanoma	Estimated incidence rates in the SEER database by 5-year age group, sex, race and calendar year were multiplied by the person-years in that stratum. The total expected number of second primary cancers was calculated by summing the expected number of second cancers in each subgroup	7
Lal [30]	Retrospective cohort study	USA	Surveillance Epidemiology and End Results (SEER)	1975–2008	Not reported for RCC	Thyroid	Cancer incidence rates specific for the strata in which person-years were distributed were multiplied by the corresponding person-years at risk to estimate the number of cancer cases expected in each stratum	7
Levi [31]	Retrospective cohort study	Switzerland	Vaud Cancer Registry	1974–1989	Not reported for RCC	Any cancer, urinary bladder and prostate	Calculation of expected numbers was based on sex-, age-, and calendar year-specific incidence rates multiplied by the observed number of person-years at risk	9
Murray [10]	Retrospective cohort study	USA	Memorial Sloan Kettering Cancer Center database	1989–2020	3066	Urinary bladder, breast, uterine, lungs, melanoma, NH lymphoma, pancreas, prostate, stomach, colorectal and thyroid	The expected number of second primaries was calculated by multiplying Surveillance, Epidemiology, and End Results Program incidence rates of renal cortical neoplasms by person-years at risk within categories of age, sex, and year of diagnosis	7
Preyer [32]	Retrospective cohort study	Austria	Tyrol and Vorarlberg Cancer Registries	1988–2005	1866	Any cancer	The expected number of second primary cancers was calculated stratified by sex, age at time of first diagnosis grouped in 5-year intervals and years of follow-up grouped in 5-year intervals as sum of PYAR multiplied by the incidence in the general population of Tyrol and Vorarlberg aggregated in one group in the respective stratum	9
Scelo [33]	Retrospective cohort study	France, Australia; Canada; Denmark; Finland; Iceland; Norway; Slovenia; Spain; Sweden; United Kingdom of Great Britain and Northern Ireland	British Columbia, Manitoba and Saskatchewan (Canada), Denmark, Finland, Iceland, New South Wales (Australia), Norway, Scotland (United Kingdom), Singapore, Slovenia, Sweden, and Zaragoza (Spain)	1943–2000		Small intestine	The expected number derived from the age-, gender-, year- and registry-specific incidence rates of first primary cancers, after excluding the expected number of small intestine neoplasms	5
Scelo [34]	Retrospective cohort study	France; Germany; Sweden; Finland; Denmark; Norway; Australia; Canada; Slovenia; Singapore; Iceland; Spain; United Kingdom of Great Britain and Northern Ireland	Cancer registries at British Columbia, Manitoba and Saskatchewan (Canada), Denmark, Finland, Iceland, New South Wales (Australia), Norway, Scotland (United Kingdom), Singapore, Slovenia, Sweden, and Zaragoza (Spain)	1943–2000	102,868	Melanoma	The expected number derived from the age-, sex-, year- and registry-specific incidence rates of first primary cancers	7
Schonfeld [35]	Retrospective cohort study	USA	Surveillance Epidemiology and End Results (SEER)	2000–2014	102,114	Thyroid	Expected numbers were obtained by multiplying incidence rates stratified by race (white/unknown, black, other), sex (male/female), attained age groups and attained calendar year by stratum-specific person-years at risk and summing across strata	7
Tabuchi [36]	Retrospective cohort study	Japan	Osaka Cancer Registry	1985–2004	Not reported for RCC	Any cancer, breast, uterine, gallbladder, lungs, esophagus, liver, pancreas, prostate, stomach, colorectal, and oropharynx	The expected number was calculated according to sex, age group, selected site of the first and second cancer, and follow-up interval	8
Zheng [37]	Retrospective cohort study	USA	Surveillance Epidemiology and End Results (SEER)	2000–2013	2486	Urinary bladder, brain, nervous system, breast, cervix, uterine, ovary, lungs, NH lymphoma, esophagus, liver, prostate, stomach, colorectal and thyroid	The expected number was based on general population rates	8
Zheng [11]	Retrospective cohort study	Sweden	Swedish Cancer Registry	1990–2015	17,587	Any cancer, renal pelvis, urinary bladder, brain, nervous system, breast, lungs, melanoma, multiple myeloma, NH lymphoma, leukemia, liver, prostate, stomach, small intestine, colorectal, thyroid, oropharynx, endocrine, and soft tissue	The expected number was obtained from the incidence of SPCs as first primary in the general population considering person-years at risk	8

### Risk of bias

The quality assessment of the studies included in this meta-analysis was performed using the Newcastle–Ottawa Scale. The risk of bias assessment was done by two reviewers. Disagreements were resolved through consensus between the pair of raters or by a third rater. Studies with a rating of 6 or higher were considered high quality. Studies with a high risk of bias (low rating, < 6) were excluded in a sensitivity analysis to evaluate their potential impact on the overall findings.

### Data synthesis

To assess the overall risk of SPCs and SPCs risk by cancer type in patients with RCC, we used the random-effect meta-analysis. We combined the extracted study-specific estimates (SIR) and corresponding 95% CIs using the Der Simonian and Laird random-effects model to calculate the pooled estimates of SIR [13].

Heterogeneity among studies was quantified using the *τ*^2^ (estimated variance of the true effect sizes across studies) and *I*^2^ statistic, which describes the percentage of total variation across studies attributable to heterogeneity rather than chance [14]. An *I*^2^ value > 50% indicated substantial heterogeneity. In the case of significant heterogeneity, the data were explored further, including meta-regression (mean age, median follow-up, and % male), subgroup analysis (geographical location and decades of follow-up), cumulative and influential analyses, to explain the heterogeneity. Publication bias was examined and visualized using a funnel plot, and the asymmetry of the plot was tested by Egger’s regression test using the R package “dmetar” [15]. When there is publication bias, we used the trim-and-fill method to assess the potential impact of publication bias on the pooled effect estimate. Sensitivity analysis, excluding studies of high risk, was also conducted. Meta-regression and Egger’s test were only performed for analyses including 10 or more studies.

Statistical analyses were performed using “meta”, “metaviz”, and “dmetar” packages on R Studio version 4.5.1, with *p* < 0.05 from 2-sided tests indicating statistical significance.

## Results

### Study selection

**Fig. 1:**
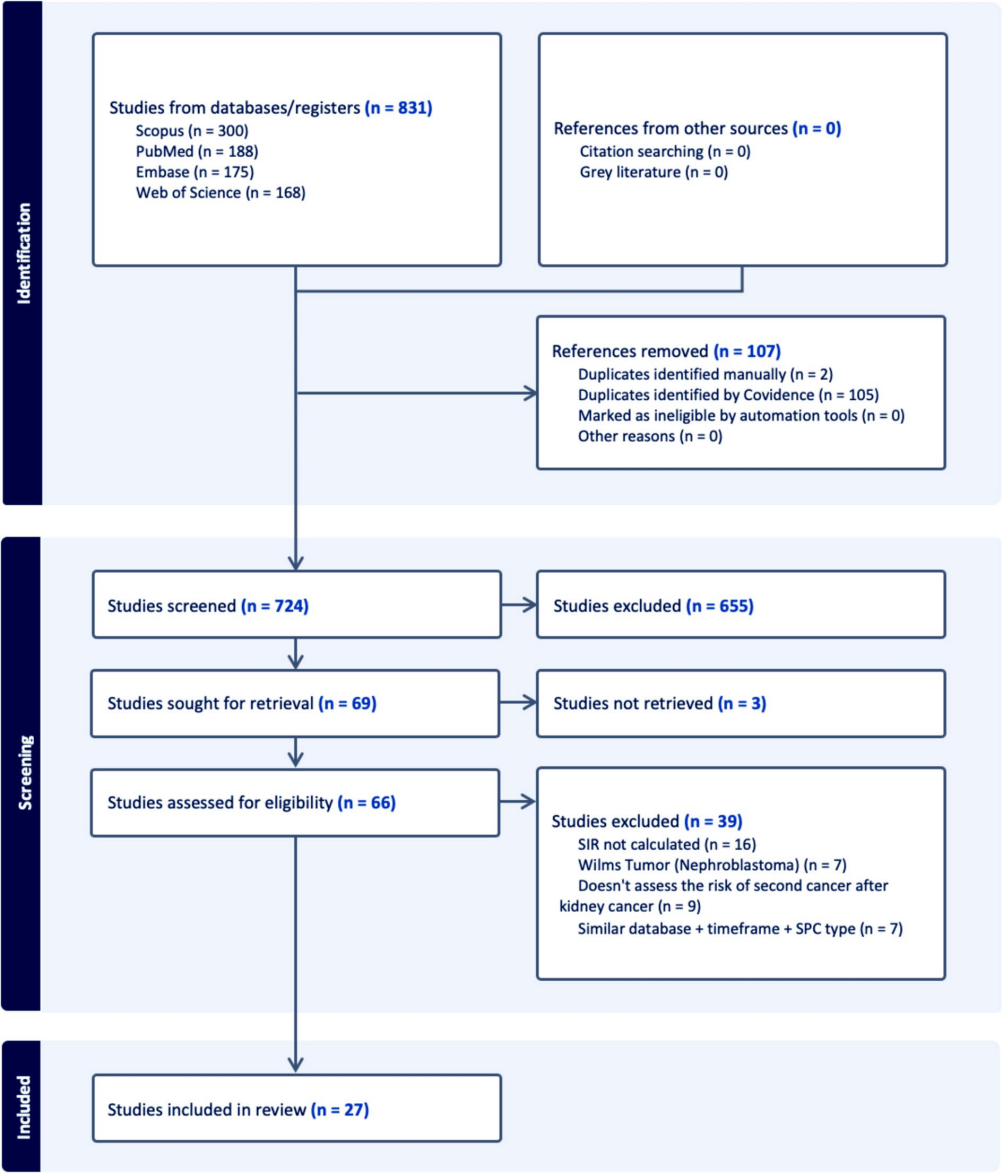
PRISMA flowchart

We initially identified 831 records through our search of the databases, of which 107 duplicates were removed, leaving 724 unique records for screening. Title and abstract screening excluded 655 records due to relevance to the study’s objective, and 69 studies were sought for full-text retrieval. Full texts were retrieved for 66 studies and were assessed for eligibility. Out of the 66 studies, 39 were excluded for the following reasons: SIR not calculated or reported (*n* = 16), Wilms tumor or nephroblastoma (*n* = 7), did not assess the risk of SPC after an RCC (*n* = 9), overlapping population or time frame, or data source/registry (*n* = 7). Finally, 27 studies [6, 7, 9–11, 16–37] met the study’s eligibility criteria and were included in the meta-analysis (Fig. 1 and Table 1).

### Characteristics of the included studies

A total of 27 studies published between 1993 and 2024 were included in the study [6, 7, 9–11, 16–37]. The studies originated from North America, Europe, and Asia. The majority of research originated from the US (*n* = 10) [6, 10, 16, 17, 19, 27, 29, 30, 35, 37], followed by Sweden (*n* = 4) [9, 11, 20, 24], Korea (*n* = 3) [21, 26, 28], Italy (*n* = 2) [22, 23], and one each from Austria [32], Denmark [7], France [25], Japan [36], Norway [18], and Switzerland [31], with two studies incorporating data from multiple countries [33, 34]. All the studies were retrospective cohort design. The follow-up period ranged from 1943 to 2019.

The studies reported the risk of SPCs for either any cancer or specific cancer types. Eleven studies reported the risk of any kind of SPC [6, 7, 11, 19, 21, 24–26, 31, 32, 36]. Site-specific SPCs were reported in the following studies: oropharynx in 7 [6, 7, 9, 11, 24, 26, 36], esophagus in 4 [9, 26, 36, 37], stomach in 9 [7, 9–11, 18, 24, 26, 36, 37], small intestine in 4 [7, 9, 11, 34], colorectal in 9 [7, 9–11, 18, 22, 24, 36, 37], pancreas in 7 [7, 9, 10, 24, 27, 28, 36], gallbladder in 2 [26, 36], liver in 7 [6, 7, 9, 11, 24, 36, 37], contralateral kidney in 4 [6, 9, 24, 25], urinary bladder in 11 [6, 7, 9, 10, 18, 22, 24, 26, 31, 37], renal pelvis in 4 [6, 7, 11, 26], leukemia in 6 [6, 7, 9, 11, 24, 26], Non-Hodgkin’s (NH) lymphoma in 9 [6, 7, 9–11, 16, 18, 24, 37], lungs/bronchial system in 10 [6, 7, 9–11, 18, 24, 26, 36, 37], endocrine system (excluding thyroid gland) in 5 [6, 7, 9, 11, 24], breast in 10 [6, 7, 9–11, 18, 24, 26, 36, 37], cervix in 3 [24, 26, 37], ovary in 3 [9, 24, 37], uterus in 5 [9, 10, 24, 36, 37], female genitalia in 3 [6, 7, 26], prostate in 10 [9–11, 17, 18, 24, 26, 31, 36, 37], male genitalia in 4 [6, 7, 11, 26], melanoma in 10 [6, 7, 9–11, 18, 24, 26, 29, 33], mesothelioma in 3 [6, 7, 20], multiple myeloma in 4 [6, 7, 9, 11], brain/nervous system in 7 [6, 7, 9, 11, 18, 24, 37], bone in 3 [6, 9, 26], soft tissue in 4 [6, 9, 11, 26], and thyroid in 8 studies [9–11, 23, 30, 35, 37].

The median Newcastle–Ottawa rating for the studies included was 8 (IQR 6–8), with low risk of bias in 21 studies (74.1%). There were over 455,907 RCC patients in the study, with the median age ranging from 59 to 66. The population characteristics and outcomes of the included studies are summarized in Table 1.

### Risk of SPCs in RCC patients

**Fig. 2:**
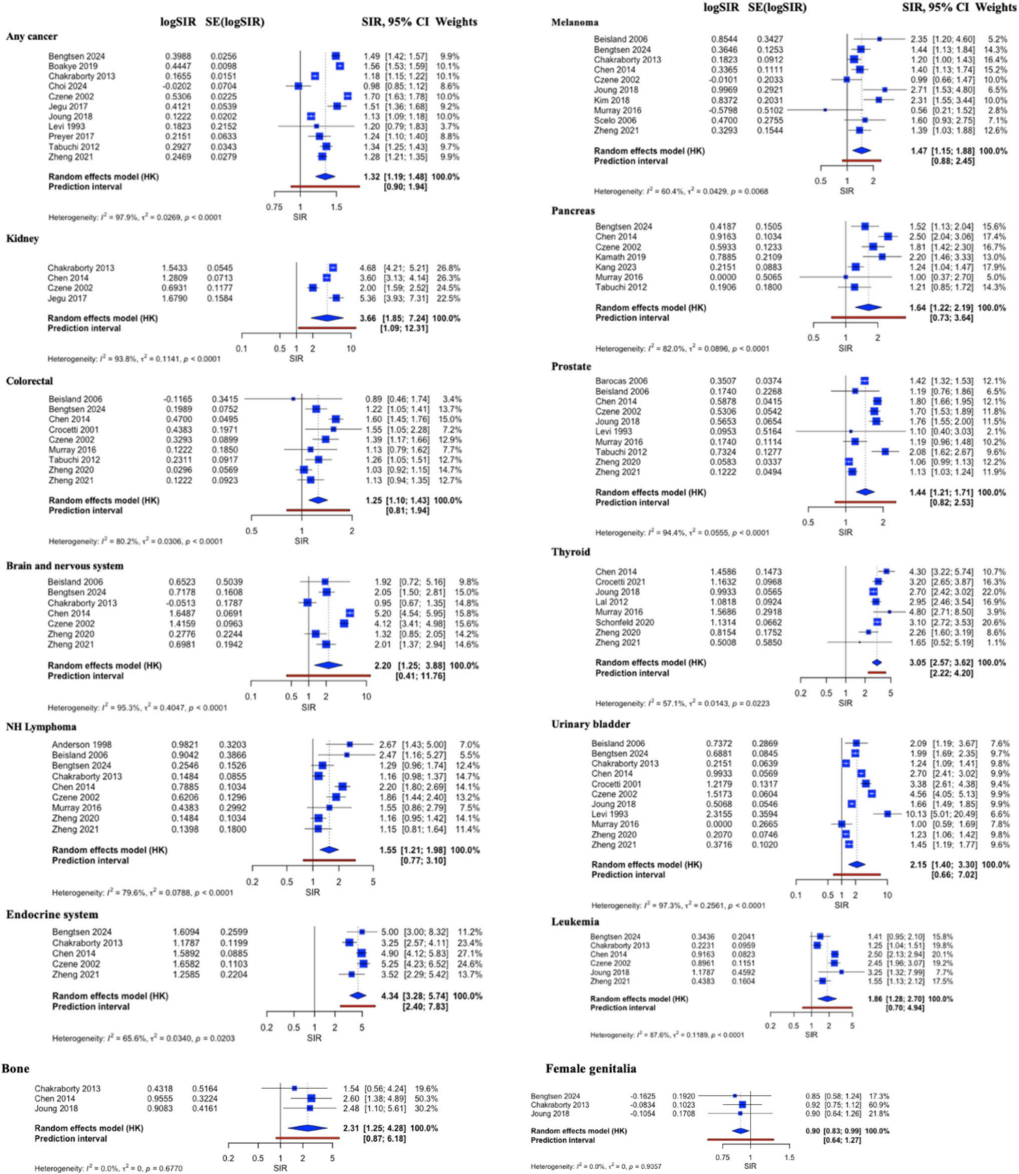
Second primary cancers with an increased risk following primary RCC (any cancer, kidney, colorectal, nervous system/brain, NH lymphoma, endocrine, bone, melanoma, pancreas, prostate, thyroid, and urinary bladder as well as second primary cancers with a decreased risk following primary RCC (female genitalia). The blue squares and their sizes represent the effect sizes and weights of the included studies, respectively. The blue diamonds and their sizes represent the pooled effect size and its 95% confidence interval, respectively. The centerline of no effect runs through the value 1. Points to the right of the centerline (> 1) indicate an increased risk, whereas points to the left of the centerline (< 1) indicate a decreased risk. The red line indicates the prediction interval

The overall risk of developing any SPCs was higher among RCC patients (pooled SIR = 1.32, CI 1.19–1.48). In addition, the risk of several SPCs was also significantly higher in patients with RCC compared with the general population’s risk of developing respective primary cancers. The risk of SPC was greatest in the endocrine system (excluding thyroid) (pooled SIR = 4.33, CI 3.28–5.74), followed by the contralateral kidney (pooled SIR = 3.68, CI 1.85–7.24), thyroid (pooled SIR = 3.05, CI 2.57–3.62), bone (pooled SIR = 2.31, CI 1.25–4.27), brain and nervous system (pooled SIR = 2.20, CI 1.25–3.88), urinary bladder (pooled SIR = 2.15, CI 1.40–3.30), small intestine (pooled SIR = 1.98, CI 1.01–3.91), leukemia (pooled SIR = 1.86, CI 1.28–2.70), pancreas (pooled SIR = 1.64, CI 1.22–2.19), NH lymphoma (pooled SIR = 1.55, CI 1.21–1.98), melanoma (pooled SIR = 1.47, CI 1.15–1.88), prostate (pooled SIR = 1.44, CI 1.21–1.71), and colorectal (pooled SIR = 1.25, CI 1.10–1.43) (Table 2, Fig. 2).

**Table 2:** Meta-analysis result showing the number of studies, pooled SIR, 95% CI, *I*^2^, and the *p*-value for the Eggers’ test by cancer site

SPC site	No. of studies	Pooled SIR	95% CI	*τ*^2^	*I*^2^	Eggers’ *p*-value
Any cancer	11	1.32	1.19–1.48*	0.027	97.9	0.378
Oropharyngeal	7	0.97	0.73–1.28	0.058	64.1	NA
Esophagus	4	1.09	0.59–2.01	0.105	81.0	NA
Stomach	9	1.27	0.87–1.85	0.175	93.5	NA
Small intestine	4	1.98	1.01–3.91*	0.100	57.3	NA
Colorectal	9	1.25	1.10–1.43*	0.031	80.2	NA
Pancreas	7	1.64	1.22–2.19*	0.090	82.0	NA
Gallbladder	2	0.94	0.01–89.75	0.204	79.6	NA
Liver	7	1.36	0.95–1.95	0.105	86.0	NA
Kidney	4	3.68	1.85–7.24*	0.114	93.8	NA
Urinary bladder	11	2.15	1.40–3.30*	0.256	97.3	0.961
Renal pelvis	4	5.07	0.40–65.01	1.820	96.1	NA
Leukemia	6	1.86	1.28–2.70*	0.119	87.6	NA
NH lymphoma	9	1.55	1.21–1.98*	0.079	79.6	NA
Lungs	10	1.23	0.98–1.53	0.057	91.2	0.969
Endocrine system	5	4.33	3.28–5.74*	0.034	65.6	NA
Breast	10	1.15	0.89–1.49	0.080	88.5	0.553
Cervix	3	0.84	0.21–3.35	0.080	75.2	NA
Ovary	3	1.20	0.71–2.02	0.015	32.6	NA
Uterine	5	1.27	0.99–1.62	0.002	5.7	NA
Female genitalia	3	0.90	0.83–0.99*	0.936	0.0	NA
Prostate	10	1.44	1.21–1.71*	0.056	94.4	0.609
Male genitalia	4	1.23	0.98–1.99	0.035	89.2	NA
Melanoma	10	1.47	1.15–1.88*	0.043	60.4	0.370
Mesothelioma	3	1.58	0.51–4.92	0.083	36.2	NA
Multiple myeloma	4	1.43	0.93–2.19	0.055	62.2	NA
Brain and nervous system	7	2.20	1.25–3.88*	0.405	95.3	NA
Bone	3	2.31	1.25–4.27*	0.677	0.0	NA
Soft tissue	4	1.55	1.00–2.38	0.002	2.5	NA
Thyroid	8	3.05	2.57–3.62*	0.014	57.1	NA

^*^Statistically significant

On the other hand, there was a decreased risk of second primary female genitalia cancer among RCC patients (pooled SIR = 0.90, CI 0.83–0.99) (Table 2 and Fig. 2). There appeared to be a potential (marginally not significant) increase in the risk of SPCs of the liver (pooled SIR = 1.36, CI 0.95–1.95), lungs (pooled SIR = 1.23, CI 0.98–1.53), uterus (pooled SIR = 1.27, CI 0.99–1.62), male genitalia (pooled SIR = 1.23, CI 0.98–1.99), multiple myeloma (pooled SIR = 1.43, CI 0.93–2.19), and soft tissues (pooled SIR = 1.55, CI 1.00–2.38). Among RCC patients, there was no difference in the risk of SPCs of the oropharynx, stomach, and breast. The risks of SPC of the esophagus, gallbladder, renal pelvis, cervix, ovary, and mesothelium were inconclusive due to wide 95% CI (Table 2 and eFigure 1, Supplement).

### Heterogeneity

There was substantial heterogeneity (*I*^2^ > 50%) in studies investigating the risk of SPC of any cancer, oropharynx, esophagus, stomach, small intestine, colorectal, pancreas, gallbladder, liver, kidney, urinary bladder, renal pelvis, NH lymphoma, lungs, endocrine system, breast, cervix, prostate, male genitalia, melanoma, multiple myeloma, brain and nervous system. Heterogeneity was low in studies examining the risk of the ovary, uterus, female genitalia, mesothelioma, bone, and soft tissue (Table 2, Fig. 2, and eFigure 2, Supplement). Univariate meta-regression analyses, however, found no statistical significance when controlling for year of publication, median age, percentage of male participants, and median follow-up duration. The heterogeneity remained high when analyses were stratified by geographical location (country) and decades of follow-up. Sensitivity analyses, including cumulative and leave-one-out analyses, showed no single study disproportionately influenced the results or heterogeneity.

### Publication bias

Visual inspection of funnel plots and Egger’s test showed no asymmetry, which indicated no publication biases were present in all meta-analyses except for one investigating the risk of SPC of the brain/nervous system. There was evidence of publication bias in the meta-analysis of SPC of the brain (Table 2 and eFigure 2, Supplement). The trim-and-fill procedure suggested that 4 studies were missing on the right side of the funnel plot for brain/nervous system meta-analysis. The unadjusted pooled SIR was 2.20 (95% CI 1.25–3.88); after imputing the missing studies, the adjusted pooled SIR was 4.12 (95% CI 2.05–8.26), indicating an increase in effect size but no change in the overall statistical significance. eFigure 3, Supplement shows the adjusted funnel plots with the imputed studies represented as unfilled circles.

## Discussion

This systematic review and meta-analysis suggest that patients diagnosed with RCC exhibit a significantly elevated risk of developing SPCs compared to the general population, especially SPCs of the contralateral kidney, thyroid, brain, nervous system, urinary bladder, small intestine, prostate, leukemia, endocrine system, non-Hodgkin lymphoma, colorectal region, pancreas, and melanoma. The SPC risk was significantly heightened in the endocrine system, thyroid, contralateral kidney, urinary bladder, bone, brain/nervous system, and small intestine, exhibiting a more than twofold increase in risk. Potential increased risks were also observed for liver, lungs, uterus, male genitalia, multiple myeloma, and soft tissues, while the analyses for esophagus, gallbladder, renal pelvis, cervix, ovary, and mesothelium were inconclusive.

Our study findings are consistent with previous studies that have reported an increased risk of SPCs following RCC [6, 7, 9–11, 18, 19, 21, 24, 26]. As a result, several possible mechanisms have been discussed. The mechanisms through which RCC is associated with other cancers may be complex and multifactorial. These associations may result from enhanced diagnostic effort, treatment effects, and shared risk factors [7]. For example, a noted explanation by Beisland et al. is that the significant risk of SPCs following RCC, especially bladder cancer, may be a result of surveillance bias, given the increased frequency of urological follow-up [18]. However, we believe this explanation might be unlikely because bladder cancer cases are not limited to the period immediately after RCC diagnosis (when monitoring is most intensive) but also emerge more than a decade later, as shown by Czene et al. [24] and Beisland et al. [18]. After the initial post-treatment period, RCC patients typically continue to undergo surveillance imaging at longer intervals, and for aggressive malignancies such as metastatic RCC (mRCC), this follow-up may be indefinite based on individualized clinical decision-making [38]. Even RCC patients with no evidence of active disease generally receive substantially more imaging during follow-up than the general population [39]. This increased frequency of imaging likely contributes to a higher rate of SPCs among RCC patients compared to the general population.

A more compelling explanation could involve the possible influence of common environmental or genetic factors. For example, Begg et al. identified smoking as a substantial factor in the increased chance of developing some SPCs [40]. The observed association between RCC and gastrointestinal cancers, such as the small intestine and pancreas, may also, at least in part, reflect common underlying risk factors, such as smoking, elevated body mass index, and high blood pressure [41, 42]. It is also plausible that other carcinogens eliminated via the kidneys might play a role in driving this association.

In addition, cancer treatments are known to result in SPC [43]. Considering that the routine therapies for RCC typically do not include chemotherapy or radiation therapy, it is unlikely that treatment-related factors substantially influence the increased risk of SPC. The immune system, such as interferon-alpha and interleukins, has been implicated in both RCC and melanoma, which might explain the increased risk of second primary melanoma among RCC patients [44]. Some research studies have indicated an elevated risk of developing a second RCC in patients with thyroid cancer [45], implying that genetic associations between RCC and thyroid cancer may contribute to the increased incidence of SPC observed in this meta-analysis.

Recent advances in systemic therapies for mRCC have markedly prolonged overall survival, which may in turn influence the observed incidence of SPCs. For example, median overall survival for mRCC has increased from approximately 12 months during the IL-2 era to over 50 months in recent trials [46]. These extended survival times provide a longer window for the development and clinical detection of SPCs, especially in the context of ongoing surveillance and follow-up care. The impact of prolonged survival is twofold: it not only increases the likelihood of SPC occurrence over time but also enhances the opportunity for their diagnosis, potentially contributing to higher reported incidences in more recent cohorts. Consequently, temporal trends in RCC treatment advances should be considered when interpreting SPC risk estimates, as improvements in survival may partially explain observed differences across studies conducted in different treatment eras.

The management of RCC patients typically entails a multidisciplinary approach, incorporating standard protocols based on prognostic criteria and the individualized needs of the patient. Given that many SPCs may be identified during follow-up, it is crucial for optimal clinical treatment to comprehend and acknowledge the potentiality and risk of SPCs in RCC patients. The findings of this meta-analysis could enhance cancer survivorship education for both patients and practitioners regarding issues that are significant to patients.

### Limitations and strengths

Our meta-analysis has several limitations. (1) There might be misclassification of cancers in a registry-based study, which might under- or overestimate the SIR. However, many of the included studies did not include synchronous SPCs diagnosed at the same time or within 6 months of the RCC diagnosis. (2) There may also be misclassification concerning the discrete sites of the SPCs. Certain studies indicated aggregated cancer sites, including the endocrine system, nervous system, male genitalia, and female genitalia. It was impossible to evaluate the risk of second cancer of organs comprising those systems at a granular level. This meta-analysis found a decreased risk of subsequent female genitalia cancer after RCC. However, it was not feasible to ascertain the precise component of the “female genitalia” as indicated by the studies included. (3) The heterogeneity among the studies was high. Although we attempted to find the factors responsible for this heterogeneity, it remained throughout the meta-analysis. This could probably be attributed to epidemiological differences between the studies, including differences in national cancer registries, proportions of histological subtypes, treatment era variations, follow-up duration, timeframes analyzed, shifts in specific cancer demographics over time, differing selection criteria, and temporospatial differences in treatment. It is noteworthy that similar significantly high heterogeneity has been reported in meta-analyses of the SPCs risk in colorectal cancer [47], breast cancer patients [48], and those with HPV-associated malignancy [49]. The high heterogeneity identified in our study warrants a cautious interpretation of the findings. Although the true magnitude remains uncertain due to heterogeneity, this meta-analysis strongly indicates a substantially higher risk of SPCs among RCC patients as reported by many of the included individual studies. We suggest that future meta-analyses should consider stratification by RCC subtypes (e.g., clear cell vs. non-clear cell). (4) There was limited data to examine the influence of RCC stage, treatment modalities (e.g., targeted therapy vs. surgery), imaging modalities, or screening frequency on SPC risk, the time between RCC and SPC, and the effect of SPCs on the overall survival of the RCC patients.

Despite these limitations, this was the first meta-analysis to comprehensively examine the pooled risk of SPCs in RCC patients and stratify the risk by the various possible second primary cancer sites.

## Conclusion

This systematic review and meta-analysis suggest that patients with RCC have a significantly higher risk of developing second primary malignancies SPCs relative to the general population. The increased risks encompass multiple organs, such as the contralateral kidney, thyroid, bone, brain/nervous system, urinary bladder, prostate, and small intestine. While the substantial heterogeneity observed across studies necessitates a cautious interpretation of the pooled estimates, the overall evidence strongly supports a heightened vigilance for SPCs in RCC survivors.

## Supplementary Information

Below is the link to the electronic supplementary material.

Supplementary file1 (DOCX 1011 KB)

## Data Availability

All the data were extracted from secondary sources and are cited in the manuscript. The datasets generated during and/or analyzed during the current study are available from the corresponding author on reasonable request.
