# Supplementary material for: Risk of developing a second primary cancer following a renal cell carcinoma: a systematic review and meta-analysis
Source: Int Urol Nephrol. 2025 Sep 18;58(4):1161–80. doi: 10.1007/s11255-025-04794-7 (PMC12999740; doi:10.1007/s11255-025-04794-7)
Supplement: Supplementary file 1 — Supplementary file1 (DOCX 1011 KB) [file 11255_2025_4794_MOESM1_ESM.docx]

**SUPPLEMENTARY INFORMATION**

**eTable 1: Keywords for study search to identify published articles on the risk of SPC among RCC patients**

| **Database** | **Search Keywords** |
| --- | --- |
| PubMed/Medline | ("Neoplasms, Multiple Primary/epidemiology"[Mesh] OR "Neoplasms, Multiple Primary/prevention and control"[Mesh] OR "Neoplasms, Second Primary/epidemiology"[Mesh] OR "Neoplasms, Second Primary/prevention and control"[Mesh] OR “Second cancer” OR “subsequent cancer” OR “second malignancies”) AND (“Carcinoma, Renal Cell"[Mesh] OR “Renal cell cancer” OR “Kidney cancer”) |
| Scopus | ( ( ( ( TITLE-ABS-KEY ( multiple AND primary AND malignancies ) ) OR ( TITLE-ABS-KEY ( second AND cancer ) ) OR ( TITLE-ABS-KEY ( second AND malignancies ) ) OR ( TITLE-ABS-KEY ( multiple AND primary AND cancer ) ) OR ( TITLE-ABS-KEY ( subsequent AND cancer ) ) ) AND ( ( TITLE-ABS-KEY ( kidney AND cancer ) ) OR ( TITLE-ABS-KEY ( kidney AND carcinoma ) ) OR ( TITLE-ABS-KEY ( renal AND cell AND cancer ) ) OR ( TITLE-ABS-KEY ( renal AND cell AND carcinoma ) ) ) ) AND ( TITLE-ABS-KEY ( risk ) ) ) AND ( TITLE-ABS-KEY ( population AND research ) ) AND ( LIMIT-TO ( SUBJAREA , "MEDI" ) ) AND ( LIMIT-TO ( DOCTYPE , "ar" ) ) AND ( LIMIT-TO ( EXACTKEYWORD , "Human" ) ) |
| Embase | ('kidney cancer'/exp OR 'cancer, kidney' OR 'carcinomatous kidney' OR 'kidney cancer' OR 'kidney carcinogenesis' OR 'kidney malignancies' OR 'kidney malignancy' OR 'malignancies of the kidney' OR 'malignancy of the kidney' OR 'malignant kidney neoplasm' OR 'malignant kidney tumor' OR 'malignant kidney tumour' OR 'malignant neoplasm of the kidney' OR 'malignant renal neoplasm' OR 'malignant renal tumor' OR 'malignant renal tumour' OR 'malignant tumor of the kidney' OR 'malignant tumour of the kidney' OR 'renal cancer' OR 'renal carcinogenesis' OR 'renal malignancies' OR 'renal malignancy' OR 'renal malignant tumor' OR 'renal malignant tumour' OR 'renal cell carcinoma'/exp) AND ('second cancer'/exp OR 'metachronous cancer' OR 'metachronous double cancer' OR 'second cancer' OR 'second cancers' OR 'second malignancies' OR 'second malignancy' OR 'second malignant neoplasm' OR 'second primary cancer' OR 'second primary cancers' OR 'second primary malignancies' OR 'second primary malignancy' OR 'subsequent neoplasm'/exp) AND ('article'/it) AND ('prospective study'/de OR 'retrospective study'/de) |
| Web of Science | ((((TS=(second cancer)) OR TS=(multiple primary malignancies)) OR TS=(multiple primary cancer)) OR TS=(second malignancies)) AND (((TS=(kidney cancer)) OR TS=(renal cell cancer)) OR TS=(renal cell carcinoma)) AND ((TS=(risk)) OR TS=(incidence)) AND (ALL=(population-based)) |


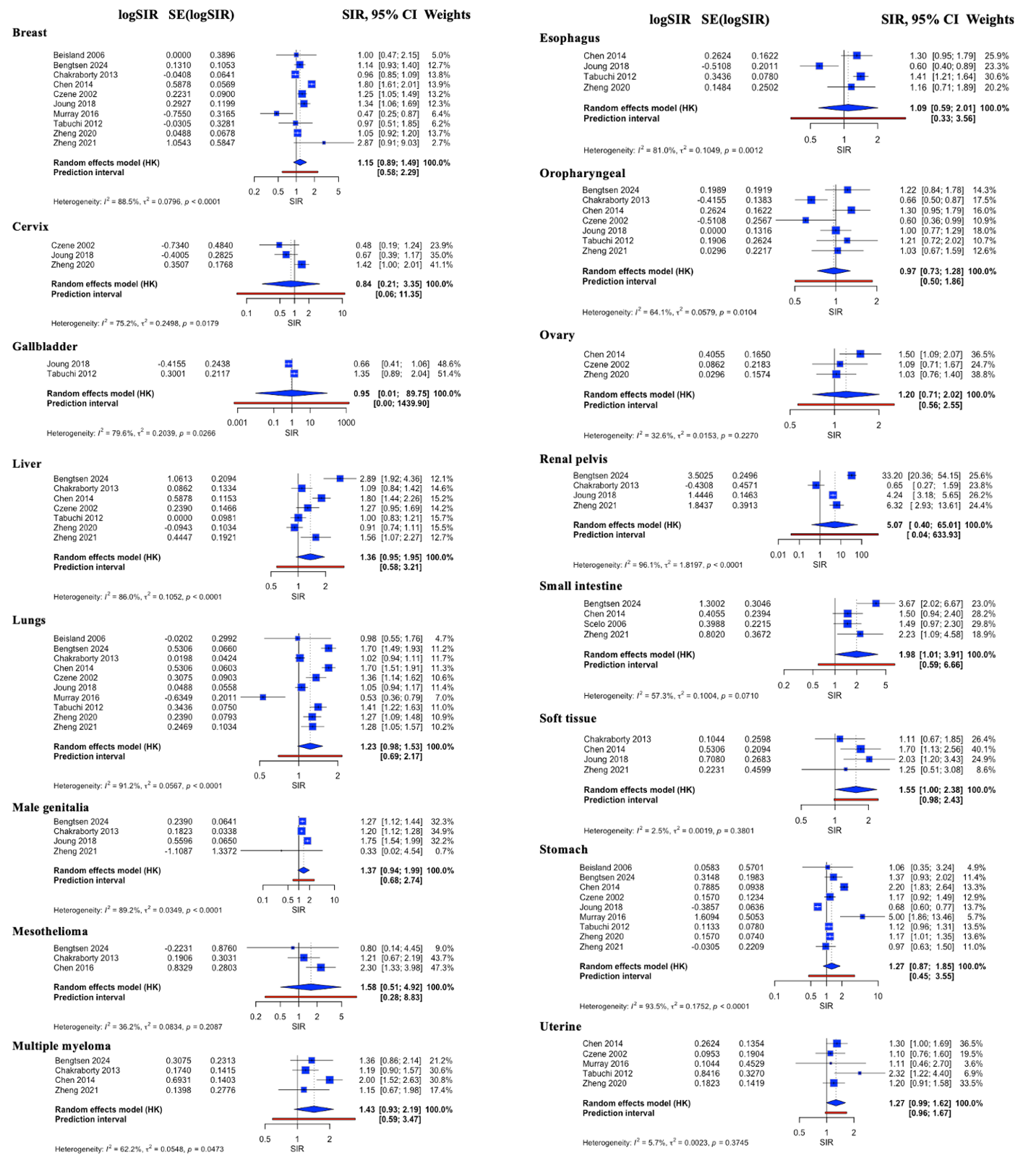


**eFigure 1. Second primary cancers in which there is inconclusive or no significant difference in the risk following primary RCC (breast, cervix, gallbladder, liver, lungs, male genitalia, mesothelioma, multiple myeloma, esophagus, oropharyngeal, ovary, renal pelvis, small intestine, soft tissue, stomach, and uterus)**


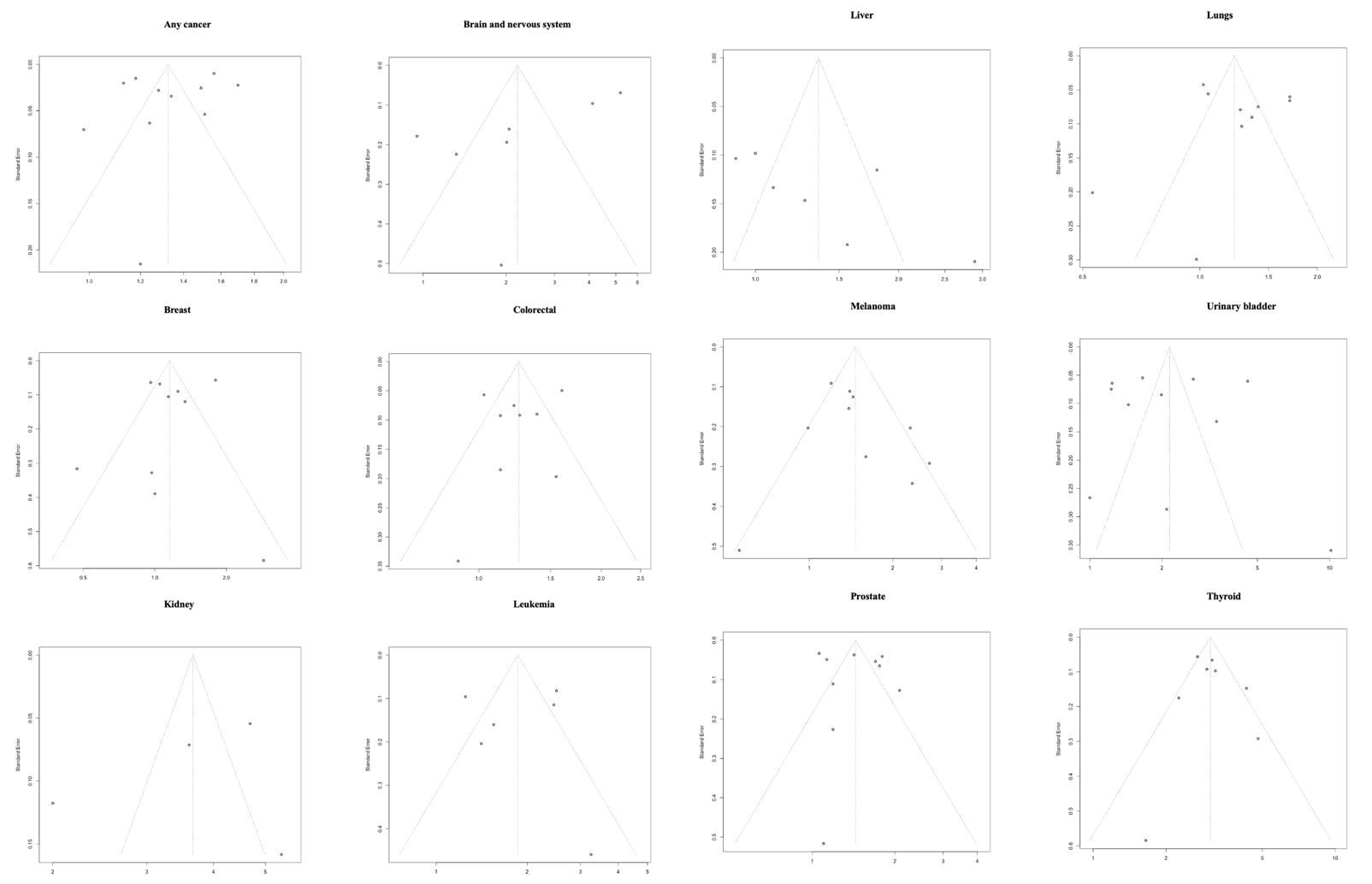


**eFigure 2. Funnel plots for selected cancers**


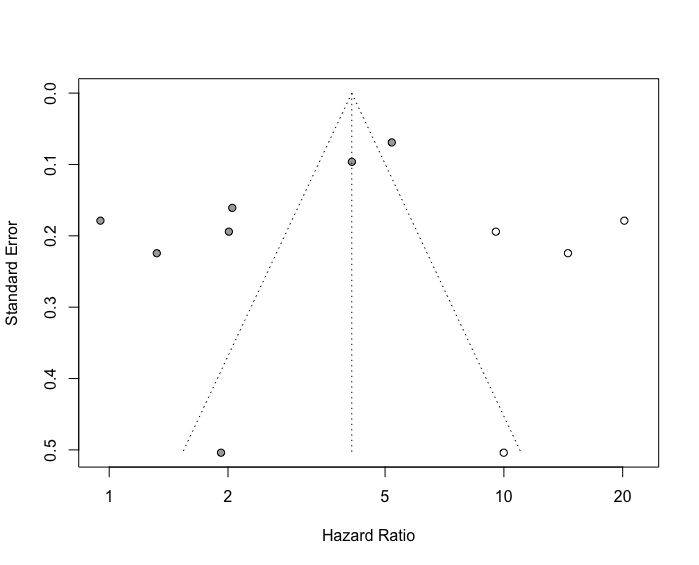


**eFigure 3. Adjusted funnel plot for meta-analysis of brain/nervous system SPC following RCC using the trim-and-fill method.**
